# Feasibility of a parent-only DBT skills group in public mental health services: a pilot implementation study

**DOI:** 10.3389/fpsyt.2026.1888780

**Published:** 2026-08-13

**Authors:** Shirel Dorman-Ilan, Ayelet Salomon, Noa Rubin, Naama de la Fontaine, Ravit Marom, Ronnie Meilik, Sapir Yona, Gal Aharon, Maya Lifshitz, Doron Gothelf, Nimrod Hertz-Palmor

**Affiliations:** 1 The Edmond and Lily Safra Children’s Hospital, Sheba Medical Center, Ramat Gan, Israel; 2 School of Psychological Sciences, Tel Aviv University, Tel Aviv, Israel; 3 Child Study Center, Yale School of Medicine, New Haven, CT, United States; 4 Intensive Care Unit for Adolescents, Geha Mental Health Center, Petah Tikva, Israel; 5 The Faculty of Medical and Health Sciences, Tel Aviv University, Tel Aviv, Israel; 6 Sagol School of Neuroscience, Tel Aviv University, Tel Aviv, Israel; 7 Medical Research Council Cognition and Brain Sciences Unit, University of Cambridge, Cambridge, United Kingdom

**Keywords:** adolescent suicidality, collective trauma, emotion dysregulation, family power struggles, parental helplessness, parent-only DBT, public mental health services, self-harm

## Abstract

**Background and objective:**

War-related mass trauma and large-scale terrorist attacks disproportionately affect clinically vulnerable adolescents, exacerbating self-harm and suicidality while burdening under-resourced public mental health systems. This study evaluated the feasibility and preliminary efficacy of a parent-only Dialectical Behavior Therapy (DBT) skills group within routine care. We hypothesized that the parent-only DBT skills group would be associated with improved parental emotion regulation, decrease helplessness and family conflict, and indirectly reduce adolescent risk behaviors.

**Methods:**

A 20-session DBT group was delivered to 41 parents of 32 adolescents aged 12–18 presenting with severe emotion dysregulation, self-harm behaviors, and/or a history of suicide attempts. Parental outcomes were assessed at baseline, post-intervention, and 3-month follow-up; adolescent clinical improvement was rated using the CGI-S 3–6 months post-treatment. Adolescent clinical severity was evaluated using the CGI-S, rated by independent clinical assessors fully blinded to participant identity, assessment time points, and duplicate cases.

**Results:**

Of the 43 parents who began the parent-only DBT skills group, 41 (95.3%) completed the intervention. Intervention participation was associated with significant reductions in parental helplessness and family power struggles. These family-level improvements coincided with clinician-rated global clinical improvement among adolescents at 3–6 months post-treatment.

**Conclusions:**

Findings support the feasibility and scalability of a targeted, parent-focused DBT adaptation. This model offers a viable clinical model for overburdened public mental health systems treating high-risk adolescents, and may be especially relevant during ongoing war and collective trauma, when clinical needs intensify while resources remain limited.

## Introduction

Difficulties in emotion regulation (ER) are increasingly conceptualized as a transdiagnostic mechanism underlying a wide range of psychiatric disorders, including depressive, anxiety, trauma-related, and personality disorders (1, 2). ER deficits are robustly associated with self-harm and suicidality (3), particularly during adolescence, a developmental period marked by a sharp rise in suicidal ideation and attempts (4). Meta-analytic findings indicate that poor ER skills heighten vulnerability to both internalizing and externalizing psychopathology, whereas effective ER functions as a protective factor against self-injurious and suicidal behaviors (5).

In Israel, these concerns have intensified in the context of recurrent military conflict, terror, and collective trauma, culminating in the mass terror attack of October 7, 2023 and the subsequent war (6). Adolescents have been exposed not only to direct and indirect threat, including rocket fire, sirens, and media coverage of violence, but also to prolonged uncertainty regarding personal safety and the safety of family members (7, 8). Many families have experienced displacement from their homes, repeated school closures, and disruptions to academic routines, lasting over two years (9, 10). Others have faced parental mobilization to military service, economic instability, or bereavement within their immediate or extended communities (11, 12). Among those not directly affected, constant exposure to traumatic content through social media and peer networks has contributed to heightened vigilance and emotional arousal (13, 14). Collectively, these conditions create sustained stress, erosion of daily structure, and reduced access to normative developmental supports, all of which are known to amplify emotional vulnerability during adolescence (15, 16). Such cumulative stressors may increase suicide risk among vulnerable adolescents by intensifying emotion dysregulation, hopelessness, social isolation, and perceived lack of safety, while simultaneously reducing access to stabilizing family, school, and community supports. Simultaneously, the public mental health system faces severe overload, characterized by long waitlists and limited access to evidence-based treatments. The convergence of increased clinical need and constrained service capacity leaves many high-risk adolescents without timely intervention (17–19).

Such conditions urge scalable and resource-efficient interventions for adolescent self-harm and suicidality. Dialectical Behavior Therapy (DBT), developed by Marsha Linehan and colleagues to target pervasive ER deficits (20), remains the primary treatment with consistent empirical support for reducing self-harm and suicidal behavior. Although originally designed for borderline personality disorder, DBT has since demonstrated efficacy across a broader diagnostic spectrum (21–23). Its transdiagnostic utility is commonly attributed to its central focus on enhancing emotion regulation (24), thereby addressing a core mechanism implicated in diverse forms of psychopathology (25).

Despite its effectiveness, standard DBT is intensive and resource-demanding. A full DBT program typically requires a team of clinicians trained in the model and includes weekly skills group training (1.5–2.5 hours), individual therapy (approximately 1 hour), between-session telephone coaching, and a therapist consultation team meeting (26). Implementing this model within public health services often necessitates substantial organizational restructuring and sustained investment in specialist training. These structural demands pose significant barriers to implementation, particularly in overburdened public systems (27, 28). In addition, suicidal and self-harming adolescents may struggle with consistent attendance, cooperation, and treatment retention due to emotional dysregulation, avoidance, family conflict, and ambivalence toward treatment. These challenges highlight the need for scalable parent-focused interventions that can strengthen the caregiving environment even when adolescents’ direct engagement is limited or unstable (29).

A parent-only intervention offers distinct clinical and practical advantages for high-risk adolescents. From a clinical perspective, suicidal and self-harming behaviors are frequently fueled by negative family dynamics, where an adolescent’s emotional dysregulation and a parent’s feelings of fear, helplessness, or criticism can trigger a destructive feedback loop (30, 31). When parents feel overwhelmed, they often resort to overcorrection, avoidance, or excessive accommodation, which unintentionally prolongs the adolescent’s crisis (32, 33). By equipping parents with skills in emotion regulation, validation, dialectical thinking, and effective boundary-setting, we can reshape the home environment to defuse family escalation (34, 35). Logistically, parents are typically more motivated and available for treatment than adolescents in crisis, who may resist or skip therapy. Consequently, a parent-only approach provides a critical avenue to improve the youth’s immediate environment, even when direct engagement with the adolescent is difficult to maintain (29).

One potential strategy to increase feasibility is to target parents directly. From an implementation perspective, parents may be more available and motivated to engage consistently in treatment than adolescents in acute crisis, who may be ambivalent, avoidant, or inconsistent in attendance. Thus, a parent-only model offers a way to intervene in the adolescent’s immediate relational context even when direct adolescent engagement is limited or unstable (29). Emerging evidence highlights the importance of parental emotion regulation, parental responses to adolescent distress, and family dynamics in the treatment of suicidal youth (30, 36). A pioneering pilot study by Berk et al. (29) evaluated a parent-only DBT-based intervention with parents of adolescents who had attempted suicide or engaged in non-suicidal self-injury. Parents reported reductions in depressive symptoms, emotion dysregulation, caregiver strain, psychiatric distress, and interpersonal sensitivity from baseline to six-month follow-up. While promising, that intervention was delivered individually, limiting scalability within public systems due to therapist time and cost requirements.

Building on this work, the present study examined the feasibility of implementing a parent-only DBT skills group within a heterogeneous public mental health setting (for a similar approach see (37). The primary aim was to evaluate whether a group-based format could be delivered within routine services and yield meaningful clinical improvement. Specifically, we assessed effects on parental emotion regulation, parental helplessness, and family power struggles or aggressive outbursts. In addition, we examined preliminary effects on adolescent suicidal and self-harm behaviors. By testing a lower-resource, parent-focused adaptation of DBT, this study sought to evaluate a scalable model for addressing adolescent suicidality in resource-constrained public systems.

To evaluate the model, the intervention was delivered to 41 parents of 32 adolescents aged 12–18 with severe emotion regulation difficulties, prior risk behaviors, recent self-harm, or suicide attempts. The program included 20 weekly 75-minute sessions across three parent groups and followed the standard DBT skill training agenda, covering mindfulness, dialectics and validation, emotion regulation, distress tolerance, and interpersonal effectiveness. In this context, dialectics refers to helping parents hold two seemingly opposing truths at the same time, such as accepting the adolescent’s emotional pain while also encouraging change and limit-setting. Feasibility and clinical outcomes were assessed longitudinally. Parents completed measures at baseline, immediately post-intervention, and at a 3-month follow-up from the last session. Youth outcomes were assessed through a senior psychiatrist’s clinical evaluation at 3–6 months follow-up.

Based on this design, we hypothesized that the parent-only DBT intervention would be associated with improvements in parental emotion regulation, decreased parental helplessness, and reductions in family power struggles and aggressive outbursts. We further hypothesized that strengthening these parent-level and family-level mechanisms would coincide with clinician-rated global clinical improvement among adolescents at follow-up, including clinical impressions of reduced risk-related distress and behaviors.

## Methods

This was a single-arm pre–post study examining the feasibility of implementing the treatment in a public mental health service setting. The Sheba Medical Center Institutional Review Board (SMC-1757-24) approved the study and waived informed consent for the retrospective analysis of anonymous data that was routinely collected for clinical purposes at the outpatient clinic. The data were originally collected as part of routine clinical group work and were later approved by the institutional review board for retrospective analysis after the groups had concluded and the data had been anonymized.

### Participants

The initial sample included 43 parents of 34 adolescents. Parents of two adolescents dropped out of the group: one parental dyad withdrew due to a significant parental conflict, and another dyad withdrew due to difficulties attending the in-person group sessions. Thus, the final study sample included 41 parents (N = 29 mothers, N = 11 fathers) of 32 adolescents (ages 12–18 years) suffering from major emotion regulation difficulties, prior risk behaviors, recent self-harm or a suicide attempt participated in the DBT parent group. Inclusion criteria included: a) child age between 12–18 years old; (b) major emotion regulation difficulties, prior risk behavior, self-harm or a suicide attempt; c) child previously received treatment as usual; however, they did not receive any additional treatment as part of the study. d) child living with the participating parent (or in shared custody, when parents were divorced); and c) at least one parent committed to consistent attendance. Exclusion criteria were a) children diagnosed with a psychotic disorder or intellectual disabilities; b) parental or familial dysfunction hindering participation (e.g., parental intellectual disabilities, substance abuse, psychotic disorder).

The adolescents mean age was 14.88 (range 12-18, SD = 1.88), and 63% were female (see **Table 1** for demographic details). Adolescents were most commonly diagnosed with depression (53%); however, diagnoses were not mutually exclusive, and 69% had at least one additional diagnosis (see **Table 2**). Additionally, 75% of adolescents reported suicidal thoughts, 47% reported current self-harm, 56% reported past self-harm, and 44% had a history of suicide attempts.

**Table 1:** Participant demographic characteristics.

Characteristic	*n*	%
Gender		
Females	20	63%
Males	11	34%
Other	1	3%
Parents’ marital status		
Single	2	6%
Married/partnered	16	50%
Divorced/separated	14	44%
Interparental conflict	4	13%
Parents’ employment status		
Both parents employed	30	94%
Single parent employed	2	6%
Mother’s educational level		
Master’s degree or higher	10	31%
Bachelor’s degree	14	44%
High school or lower	7	22%
N/A	1	3%
Father’s educational level		
Master’s degree or higher	10	31%
Bachelor’s degree	9	28%
High school or lower	10	31%
N/A	1	3%
Single mothers	2	6%

*N=*32. Participants’ mean age was 14.88 ± 1.88 years old (*Range = 12-18*).

**Table 2:** Patient diagnoses at baseline.

Diagnoses	*n*	%
Depressive disorders	17	53%
Anxiety disorders	10	31%
ADHD	15	47%
PTSD	4	13%
OCRD	2	6%
Behavioral disorders	3	9%
Suicidal thoughts	24	75%
Current self-harm	15	47%
Past self-harm	18	56%
Suicide attempt	14	44%
ASD	3	9%
Personality disorder	2	6%
Other	7	22%
Comorbidity	22	69%

*N=*32. Average diagnoses were 2.09 ± 0.96 (*Range = 1-4*).

### Procedure

Following a diagnostic interview by a senior psychiatrist or psychologist, based on the DSM-5 diagnostic criteria, parents of youth diagnosed with recent risk behavior, self-harm or a suicide attempt were referred to the DBT-based parent group treatment. All three parent groups were recruited and treated after the October 7 attack and during the ongoing war. The first group began closest to the onset of the war, running from 12/20/2023-04/10/2024; the second group ran from 11/06/2024-03/26/2025; and the third group ran from 01/08/2025-06/11/2025. No group was ongoing at the time of the October 7 attack. Participants then underwent a baseline evaluation and interview by an expert clinical psychologist or psychotherapist to confirm eligibility for the treatment. They received psychoeducation on child emotion regulation disorders and the DBT protocol (35, 38). After providing verbal consent, parents completed baseline self-report measures via a secure link (Time 1/Pre). The DBT group intervention followed the original protocol (34) but was shortened to make it more suitable for the public service (see description of DBT-based parent group treatment below); adherence monitoring is described below. Parents also completed outcome assessments at the end of the intervention (Time 2/Post) and at a 3- months follow-up (Time 3/Follow-Up). Youth completed a psychiatric assessment by a senior psychiatrist at a 3–6-months follow-up visit.

### Description of DBT-based parent group treatment

The intervention consists of 20 weekly group sessions, 1.25 hours in length. Each group included 10–15 parents. Both parents were encouraged to take part in the intervention. However, the participation of only one parent was permitted if both parents were unable to attend. Sessions were structured using the standard agenda for DBT skills training: (a) mindfulness practice, (b) dialectics and validation, (c) emotion regulation, (d) distress tolerance, (e) interpersonal effectiveness (see **Supplementary Table 1** in the **Supplementary Materials** for further elaboration).

Treatment adherence was monitored through transcript-based review and ongoing supervision. All group sessions were transcribed, enabling detailed review of session content. Facilitators reviewed the transcripts to verify that the planned DBT modules and specific skills were delivered as intended, to identify any deviations from the protocol, and to discuss implementation challenges. These issues were further reviewed in post-session facilitator meetings and in ongoing supervision with a senior clinical psychologist, in order to support consistency across groups and facilitators. No formal standardized fidelity checklist or independent fidelity ratings were used in this pilot study.

### Measures

The following self-report scales were administered to the parents at the above mentioned 3 time points:

#### Difficulties in parental emotional regulation scale

Difficulties in parental emotional regulation scale (DERS) (39) is a 41-item self-report measure developed to assess difficulties in emotion regulation. Parents are asked to rate the frequency in which they experienced distressing feelings or behaviors on a 5-point Likert-type scale (1=‘almost never’, 0-10% of the time, 5=‘almost always’, 91-100% of the time). Higher scores represent greater emotion regulation difficulty.

#### Family power struggles questionnaire

Family power struggles questionnaire (40) is a self-report questionnaire used to assess the frequency and characteristics of situations involving a power struggle between parents and children. It includes 16 items that query three types of power struggles: struggles involving arguments, resistance and violence (items 1–2, 5, 13–15), struggles involving clinging, dependence and emotional blackmail (items 3–4, 6–7, 10, 16) and struggles involving distancing or separating from the parent (items 8–9, 11–12). Items are scored on a 5-point Likert-type scale (0=‘never’; 5=‘very often’) and are averaged to derive total scores. Higher scores represent greater frequency of power struggles between child and parents.

#### Parental sense of helplessness questionnaire

Parental sense of helplessness questionnaire (41) is a 18-item instrument that assesses parental sense of helplessness in response to a child’s verbal or physical aggression (items 4, 8–11, 16, 18) and/or the child’s disrespectful behavior (items 2–3, 5–7, 13–15). Items are scored on a 6-point Likert-type scale (0=‘not at all’; 6=‘very much’) and are averaged to derive total scores. Higher scores represent a greater sense of parental helplessness.

#### Emotion regulation questionnaire

Emotion regulation questionnaire (ERQ) (42) is a 10-item self-report measure of how frequently people use two emotion regulation strategies: cognitive reappraisal (e.g., “When I want to feel less negative emotion, I change the way I’m thinking about the situation”) and expressive suppression (e.g., “I keep my emotions to myself”). All items are answered on a 7-point Likert scale, ranging from 1 (strongly disagree) to 7 (strongly agree), with higher scores indicating higher usage of that strategy.

#### Parental reflective functioning questionnaire

Parental Reflective Functioning Questionnaire (PRFQ) (43) is a 18-item self-report measure that assesses parental reflective functioning or mentalizing, that is, the capacity to treat the infant as a psychological agent. Items are scored on a 7-point Likert-type scale (1=‘strongly disagree’; 7=‘strongly agree’). Items 11 and 18 are reverse coded, and the subscale scores are the means of the items. The instrument includes three subscales: (a) Pre-Mentalizing Modes of Mental States (PM), with higher scores indicating greater difficulty with understanding and interpreting a child’s mental experience (e.g., “My son cries in front of strangers to embarrass me”); (b) Certainty about Mental States (CMS), with higher scores reflecting more certainty about what a child is thinking, feeling, or intends to do (e.g., “I always know why my child behaves the way he does”); and (c) Interest and Curiosity in Mental States (IC), with higher scores reflecting a higher level of parents’ interest in their children’s inner experiences (e.g., “I like to think of the reasons behind the way my child feels and behaves”). Optimal parental reflective functioning levels are expressed by higher scores on the IC and CMS subscales and by lower scores on the PM subscale.

#### Strengths and difficulties questionnaire

Strengths and Difficulties Questionnaire (SDQ) (44) is a widely used parent-report measure of child emotional and behavioral functioning. The questionnaire consists of 25 items across five subscales: emotional symptoms, conduct problems, hyperactivity/inattention, peer problems, and prosocial behavior. Items are rated on a 3-point scale (0–2). A total difficulties score is calculated from the four problem subscales, with higher scores indicating greater emotional and behavioral difficulties. The extended version of the SDQ includes an impact supplement assessing the perceived impact of the child’s difficulties on distress and daily functioning.

#### Clinician-rated adolescent clinical severity

Changes in adolescent clinical severity were examined using the Clinical Global Impression–Severity scale (CGI-S) (45, 46). The CGI-S is a widely used clinician-rated measure of overall illness severity, rated on a 7-point scale ranging from 1 (“normal, not at all ill”) to 7 (“among the most extremely ill”). In the present study, the CGI-S served as the primary adolescent clinical outcome because it provides a transdiagnostic index of overall clinical severity and is suitable for heterogeneous clinical presentations. Ratings were based on standardized clinical summaries derived from medical records and were completed by independent clinical assessors who were blinded to participant identity and assessment time point.

### Statistical analysis

To examine pre- to post- to follow-up changes in parental-reported outcomes, we fitted linear mixed-effects models separately for each outcome, with the outcome as the dependent variable. Time point was modeled as a categorical fixed effect, with post-treatment and follow-up contrasts specified relative to baseline, allowing for non-linear change trajectories. Participant identity was included as a random intercept to account for within-subject dependence due to repeated measurements. This approach accommodates unbalanced data under a missing-at-random assumption and preserves within-person estimation, enabling inclusion of all available observations without imputation, which was considered inappropriate given the expected non-linearity and heterogeneity of symptom change.

As sensitivity analyses, we first refit all models after excluding the pilot group, which received a slightly modified protocol. To further assess robustness given the modest sample size, we conducted a leave-one-group-out analysis, repeating the models three additional times, each time excluding one non-pilot treatment group, and examined whether the overall pattern of effects was preserved.

Finally, we examined changes in clinician-rated severity using the Clinical Global Impression – Severity scale (CGI-S), which served as the primary clinical outcome due to its widespread use as a transdiagnostic indicator of overall illness severity and its sensitivity to clinical change across heterogeneous presentations. CGI-S ratings were provided by three independent psychiatrists and clinical psychologists in the final stages of residency (authors RM, SY and GA), supervised by an experienced expert clinical psychologist (author SD-I), based on standardized clinical summaries derived from medical records. Assessors were blinded to participant identity and assessment time point and were unaware that multiple summaries could pertain to the same individual. Following an initial calibration phase in which assessors jointly reviewed cases to align severity anchors, all subsequent ratings were conducted independently, yielding three ratings per case. Inter-rater reliability was evaluated using the intraclass correlation coefficient (ICC). CGI-S change over time was then analyzed using the same mixed-effects modeling strategy described above.

P-values were adjusted for multiple comparisons using the False Discovery Rate (FDR) correction (47). All reported p-values are adjusted for false discoveries across all outcome models.

## Results

### Treatment feasibility

Of the 43 parents who began the parent-only DBT skills group, 41 completed the intervention, reflecting a high treatment completion rate of 95.3%. The protocol was delivered as a 20-session parent-only group within routine public mental health care, supporting the feasibility of implementing a streamlined DBT-based intervention in this setting.

### Parental-report

Parental report indicated significant reductions in parental helplessness at post-treatment (B=−0.59, 95% CI [−0.90, −0.28], p_adjusted_=.005) and follow-up (B=−0.61, 95% CI [−0.92, −0.29], p_adj_=.005), as well as a significant reduction in parental struggles at post-treatment (B=−0.38, 95% CI [−0.58, −0.18], p_adj_=.005) and follow up (B=−0.28, 95% CI [−0.49, −0.08], p_adj_=.041). Specifically, there were reductions in struggles involving arguments, resistance and violence post treatment (B=−0.32, 95% CI [−0.57, −0.08], p_adj_=.041), but not at follow-up; in struggles involving clinging, dependence and emotional blackmail at post (B=−0.45, 95% CI [−0.69, −0.21], p_adj_=.005) and follow-up (B=−0.31, 95% CI [−0.59, −0.06], p_adj_=.045); and in struggles involving distancing or separating from the parent at post (B=−0.31, 95% CI [−0.53, −0.09], p_adj_=.039) and follow up (B=−0.28, 95% CI [−0.51, −0.06], p_adj_=.045).

Parental reappraisal, assessed using the ERQ re-evaluation subscale, increased significantly at post-treatment (B = 0.58, 95% CI [0.22, 0.95], p_adj_ =.015), but this effect was not maintained at follow-up. Total difficulties on the SDQ reduced at post-treatment (B=−2.17, 95% CI [−3.80, −0.53], p_adj_ p=.043) and follow-up (B=−2.67, 95% CI [−4.33, −1.01], p=.015). Exploratory subscale analyses indicated pre- to follow-up improvements in hyperactivity, conduct problems, and prosocial behavior in the expected directions, though these effects did not remain significant after correction, except for reduction in conduct behaviors at follow-up (B=−0.86, 95% CI [−1.55, −0.18], p_adj_=.045) and an increase in prosocial behavior at post-treatment (B = 0.96, 95% CI [0.25, 1.67], p_adj_=.041).

Parental impulsivity, measured with the DERS impulsivity subscale, showed reductions from baseline to post-treatment and follow-up; however, these effects did not survive adjustment for multiple comparisons (post-treatment: B=−1.56, 95% CI [−2.92, −0.20], p_unadjusted_=.025, p_adj_ =.07; follow-up: B=−1.47, 95% CI [−2.85, −0.09], p_unadjusted_ =.036, p_adj_ =.10). The PRFQ three mentalization subscales did not show any change from pre- to post-treatment or to follow-up.

Descriptive statistics of pre- post-treatment and follow-up clinical outcomes are summarized in **Table 3**; Mixed-effects regressions of the post-treatment and follow-up clinical outcomes are summarized in **Table 4**.

**Table 3:** Descriptive statistics of pre-, post-treatment and follow-up clinical outcomes.

Var	Pre summary	Post summary	Follow up summary
Parental helplessness	3.10 (1.12)	2.53 (0.84)	2.49 (0.93)
Power struggles	2.66 (0.73)	2.25 (0.68)	2.35 (0.66)
Power struggles - arguments	2.63 (0.86)	2.24 (0.84)	2.35 (0.73)
Power struggles - clinging	2.89 (0.82)	2.46 (0.75)	2.60 (0.80)
Power struggles - distancing	2.28 (0.78)	1.95 (0.64)	1.97 (0.73)
DERS - total score	84.54 (17.72)	80.68 (16.88)	82.43 (17.94)
DERS - nonacceptance emotional response	14.60 (5.87)	13.52 (5.83)	13.38 (5.55)
DERS - difficulty engaging	14.00 (4.65)	13.80 (4.50)	14.21 (4.71)
DERS - impulse control difficulty	13.12 (4.74)	11.84 (4.07)	11.88 (4.97)
DERS - lack of emotional awareness	15.02 (3.83)	14.61 (2.88)	14.38 (3.64)
DERS - limited access	16.04 (6.45)	15.43 (5.25)	16.43 (5.42)
DERS - lack of emotional clarity	11.42 (2.71)	11.48 (1.96)	12.14 (1.84)
ERQ - re-evaluation	4.63 (1.08)	5.19 (0.99)	4.94 (1.14)
ERQ - expressive suppression	3.04 (1.23)	3.30 (1.12)	3.17 (1.43)
PRFQ - pre-mentalizing modes	2.49 (0.88)	2.39 (0.88)	2.38 (0.87)
PRFQ - certainty about mental states	4.00 (1.12)	3.96 (0.95)	4.11 (0.71)
PRFQ - interest and curiosity in mental states	5.02 (0.86)	5.12 (0.75)	5.06 (0.80)
SDQ - total score	19.92 (6.28)	17.74 (7.06)	17.27 (5.30)
SDQ - emotional problems	5.08 (2.60)	4.59 (2.47)	4.69 (2.63)
SDQ - peer relationship problems	4.42 (2.06)	3.81 (2.43)	4.04 (2.13)
SDQ - hyperactivity/Inattention	5.97 (2.49)	5.59 (2.66)	5.27 (2.16)
SDQ - conduct problems	4.44 (2.69)	3.74 (2.77)	3.27 (2.24)
SDQ - prosocial behavior	5.97 (1.98)	6.85 (1.97)	6.58 (2.08)

**Table 4:** Mixed-effects regressions of post-treatment and follow-up clinical outcomes.

Var	Post	Follow-up
Parental helplessness	-0.59 (-0.90, -0.28)***	-0.61 (-0.92, -0.29)***
Power struggles	-0.38 (-0.58, -0.18)***	-0.28 (-0.49, -0.08)*
Power struggles - arguments	-0.33 (-0.57, -0.08)*	-0.22 (-0.46, 0.03)
Power struggles - clinging	-0.45 (-0.69, -0.21)***	-0.31 (-0.56, -0.07)*
Power struggles - distancing	-0.31 (-0.53, -0.09)*	-0.28 (-0.51, -0.06)*
DERS - total score	-5.07 (-10.13, -0.02)	-2.98 (-8.09, 2.13)
DERS - nonacceptance emotional response	-1.30 (-3.03, 0.42)	-1.23 (-2.98, 0.51)
DERS - difficulty engaging	-0.17 (-1.68, 1.35)	0.12 (-1.41, 1.66)
DERS - impulse control difficulty	-1.56 (-2.92, -0.20)	-1.47 (-2.85, -0.09)
DERS - lack of emotional awareness	-0.56 (-1.77, 0.64)	-0.68 (-1.90, 0.53)
DERS - limited access	-0.81 (-2.65, 1.04)	0.20 (-1.67, 2.07)
DERS - lack of emotional clarity	0.09 (-0.68, 0.86)	0.76 (-0.02, 1.54)
ERQ - re-evaluation	0.58 (0.22, 0.95)*	0.33 (-0.03, 0.70)
ERQ - expressive suppression	0.18 (-0.23, 0.60)	0.08 (-0.34, 0.50)
PRFQ - pre-mentalizing modes	-0.15 (-0.41, 0.12)	-0.15 (-0.42, 0.12)
PRFQ - certainty about mental states	-0.03 (-0.39, 0.34)	0.11 (-0.26, 0.49)
PRFQ - interest and curiosity in mental states	0.09 (-0.18, 0.37)	0.04 (-0.24, 0.32)
SDQ - total score	-2.17 (-3.80, -0.53)*	-2.67 (-4.33, -1.01)*
SDQ - emotional problems	-0.68 (-1.49, 0.12)	-0.70 (-1.52, 0.11)
SDQ - peer relationship problems	-0.58 (-1.30, 0.14)	-0.33 (-1.06, 0.40)
SDQ - hyperactivity/Inattention	-0.36 (-0.97, 0.24)	-0.75 (-1.36, -0.13)
SDQ - conduct problems	-0.52 (-1.19, 0.15)	-0.86 (-1.55, -0.18)*
SDQ - prosocial behavior	0.96 (0.25, 1.67)*	0.73 (0.01, 1.44)

Values are β (95% CI). Significance indicators reflect FDR-adjusted p-values (* pFDR <.05, ** pFDR <.01, *** pFDR <.005).Bold values indicate statistically significant effects following FDR correction (adjusted p < .05). Values represent unstandardized regression coefficients with 95% confidence intervals.

### Clinical global impression

**Figure 1:**
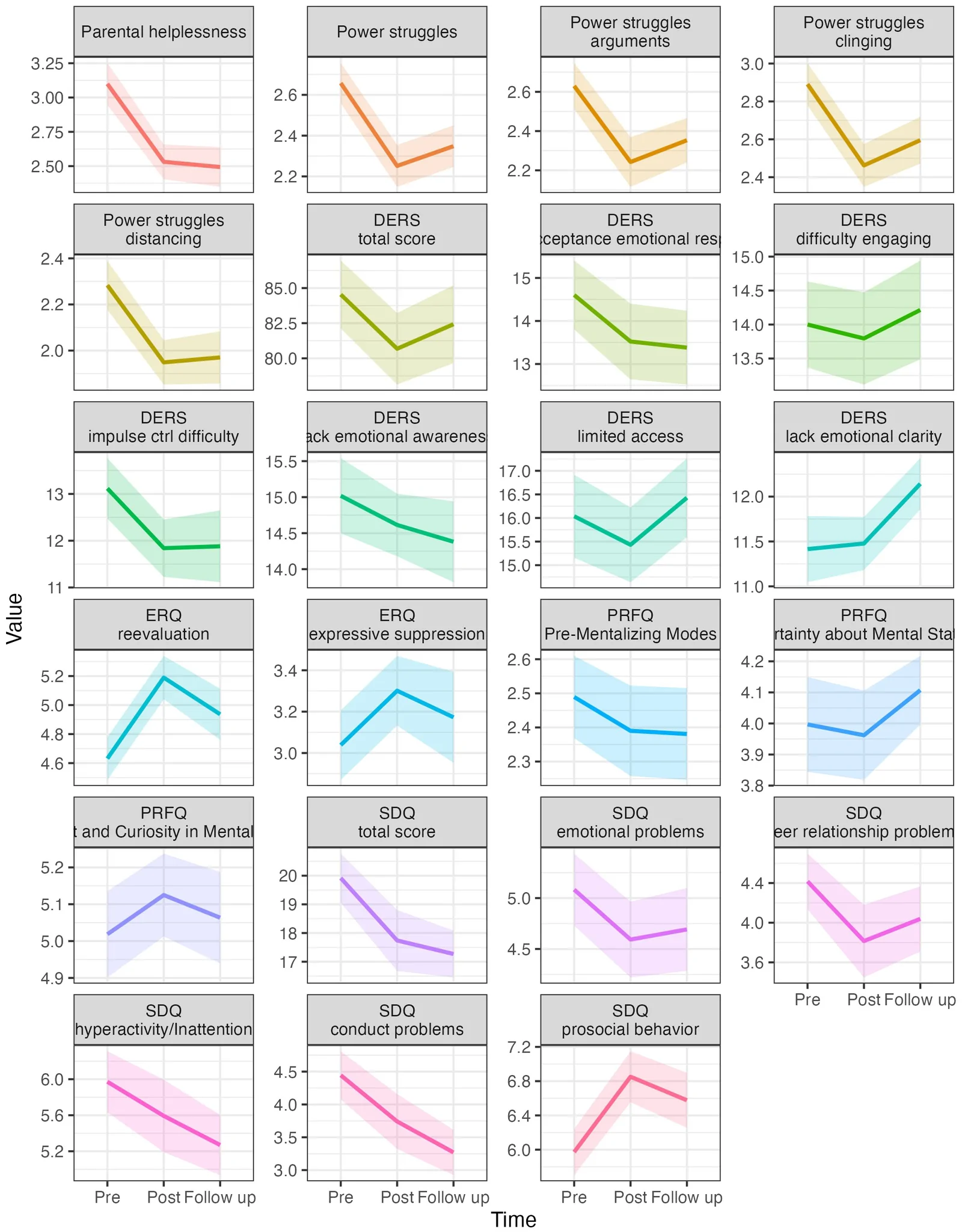
Changes in parental and adolescent clinical outcome measures from baseline (Pre) to post-intervention (Post) and 3-month follow-up (Follow-up). Lines represent estimated mean scores at each assessment point, and shaded areas represent 95% confidence intervals.

Inter-rater reliability for CGI-S ratings was excellent (ICC = 0.86, 95% CI [0.78, 0.91]), indicating high agreement among blinded clinical assessors. At baseline, mean CGI-S scores were 5.33 ± 0.93, corresponding to a markedly ill clinical presentation. At post-treatment, severity decreased to 4.83 ± 0.82, though this change was marginal (B=−0.50, 95% CI [−1.04, 0.03], p=.07). At follow-up, CGI-S scores further decreased to 4.48 ± 1.17, with a significant reduction relative to baseline (B=−0.83, 95% CI [−1.38, −0.30], p=.004), corresponding to a shift toward moderately ill severity levels. Overall, the main parental-report findings indicated the strongest and most sustained improvements in parental helplessness, with additional improvements in family power struggles and reappraisal, as illustrated in **Figure 1**.

## Discussion

The present study evaluated the feasibility and preliminary effectiveness of a parent-only DBT group intervention within a resource-limited public health setting. Consistent with our hypotheses, participation in the intervention was associated with meaningful improvements in key parental processes, most notably reductions in parental helplessness and family struggles, as well as improvements in adolescent clinical symptoms. In light of the urgent need for scalable interventions for suicidal and self-harming adolescents, particularly in the wake of the collective trauma following October 7th terror attack, these preliminary findings suggest that targeting parental factors may represent a promising clinical pathway. However, given the single-arm design, these associations should be interpreted cautiously and cannot establish causal effects or mechanisms of change.

One of the most robust findings of the study was the significant and sustained reduction in parental helplessness observed at both post-treatment and at follow-up. Parental helplessness has been identified as a core mechanism maintaining coercive family cycles, emotional escalation, and ineffective responses to adolescent emotion regulation difficulties, self-harm and suicidal crises (31, 32). FDBT conceptualizes helplessness as reflecting deficits in emotion regulation, limited access to effective coping strategies, and reduced confidence in one’s capacity to influence emotionally charged interpersonal situations (48, 49). The observed reductions may suggest that when parents feel more capable of managing crises, family interactions could become less emotionally escalated, potentially contributing to reductions in aggressive outbursts and struggles. However, this interpretation remains hypothetical, as the present study did not formally test mediation or temporal mechanisms linking changes in parental helplessness to changes in family conflict or adolescent clinical severity. The stronger and more sustained reduction in parental helplessness, relative to the less stable change in family power struggles, may be understood in light of the parent-focused nature of the intervention. Parent-only DBT directly targets parents’ emotion regulation, distress tolerance, validation, and capacity to respond more effectively during crises, all of which may enhance parents’ subjective sense of agency and reduce helplessness (29, 50). In contrast, family power struggles reflect reciprocal and often entrenched interactional patterns that depend not only on parental skill acquisition but also on adolescent responses and broader family dynamics (31). Thus, while parents may relatively quickly experience themselves as less overwhelmed and more capable, changes in conflictual family patterns may require longer consolidation and repeated practice across the family system (31, 33). Future controlled studies should examine whether reductions in parental helplessness mediate subsequent changes in family conflict, adolescent behavioral resistance, and clinician-rated adolescent severity.

The stronger and more sustained reduction in parental helplessness, relative to the less stable change in family power struggles, may be understood in light of the parent-focused nature of the intervention. Parent-only DBT directly targets parents’ emotion regulation, distress tolerance, validation, and capacity to respond more effectively during crises, all of which may enhance parents’ subjective sense of agency and reduce helplessness (29, 50). In contrast, family power struggles reflect reciprocal and often entrenched interactional patterns that depend not only on parental skill acquisition but also on adolescent responses and broader family dynamics (31). In this context, the concept of psychological reactance may offer an additional explanatory framework (51). As parents begin to respond differently, set limits more consistently, or interrupt familiar escalation cycles, adolescents may initially experience these changes as a threat to autonomy or control and respond with increased behavioral resistance. Thus, while parents may relatively quickly experience themselves as less overwhelmed and more capable, changes in conflictual family patterns may require longer consolidation, repeated practice, and gradual adaptation across the family system (31, 33).

The pattern of change in emotion regulation strategies is also clinically meaningful. The increased use of cognitive reappraisal, combined with the maintenance of suppression, is consistent with contemporary models emphasizing flexibility in emotion regulation rather than the uniform superiority of one strategy across contexts (52, 53). This shift suggests that parents may have begun to adopt more adaptive regulatory strategies while retaining some level of suppression, which may still serve a functional role in specific high-intensity or crisis situations. Importantly, reappraisal is generally considered more adaptive over the long term, as it involves modifying the meaning of an emotional situation rather than merely inhibiting emotional expression (54, 55). Thus, the observed pattern may reflect a clinically meaningful broadening of parents’ regulatory repertoire, underscoring the importance of fostering flexible emotion regulation strategies instead of simply favoring one strategy over another.

Beyond changes in specific components, we observed a decrease in overall clinical severity, reflected in the significant reduction in CGI-S scores as rated independently by three clinicians blinded to identifying details in the medical files. As a widely used global measure of illness severity, each point in the CGI-S reflects a qualitatively distinct level of clinical functioning, ranging from “not at all ill” to “among the most extremely ill patients.” Importantly, the approximately one-point reduction observed in the present study corresponds to a shift from “markedly ill” to “moderately ill.” Although this does not indicate remission, it suggests a meaningful reduction in symptom burden and impairment that would likely be noticeable to clinicians in routine follow-up assessments, with adolescents presenting as less impaired and requiring a lower level of clinical concern. In the context of a complex, highly comorbid population treated in a public mental health service, such changes are considered clinically significant.

Interestingly, the absence of significant improvement in DERS scores, combined with the deterioration observed at follow-up, may indicate that difficulties in emotion regulation are less amenable to short-term change and require continued reinforcement beyond the active treatment phase. This highlights the need for follow-up booster sessions or check-ins to help maintain treatment gains and support parents in their ongoing efforts to regulate emotions and manage family dynamics (2, 29).

Several limitations should be noted. This was a small pilot study without a control group, limiting causal inference and the ability to separate intervention effects from spontaneous recovery or concurrent treatment as usual. In addition, several outcomes relied on parent report, which may be influenced by distress, expectations, or increased awareness (29). To mitigate this, a major methodological strength was our use of independent assessors rating clinical severity (CGI-S) under a robust, multi-layered blind (unaware of participant identities, time points, or duplicate cases). This rigorous design significantly reduced potential rater bias. Although independent clinician-rated CGI-S scores strengthened the assessment, future studies should incorporate adolescent self-report and objective observational measures. Another significant limitation is the sample’s diagnostic diversity. The clinical heterogeneity of the sample reflects the realities of public mental health services, where comorbidity is common. However, because the sample size precluded subgroup analyses, it remains unclear whether the observed improvements generalized across diagnostic presentations or were primarily attributable to specific subgroups. Future studies with larger samples should examine potential moderators of treatment response. We also examined multiple outcomes in a relatively small sample, limiting statistical power once corrections for multiple comparisons were applied. While we report marginal findings to inform future studies and identify effects that may warrant examination in larger samples, these results should be interpreted cautiously. Several secondary outcomes did not survive correction for multiple comparisons and should therefore be considered exploratory pending replication. Finally, although treatment adherence was supported by transcript-based session review, post-session facilitator meetings, and ongoing supervision, the study did not include a formal standardized fidelity checklist or independent fidelity ratings. Future trials should include systematic fidelity assessment to better evaluate the consistency of intervention delivery across facilitators and clinical settings.

Furthermore, generalizability to lower-resource public health settings or low-and-middle-income countries (LMICs) may be limited. This trial was conducted at a highly resourced, university-affiliated tertiary center with clinical psychologists receiving weekly expert supervision. Replicating this model requires specific minimum conditions: facilitators trained in foundational DBT, structured weekly peer or expert supervision, and a stable physical venue. Moreover, the 20-session, 75-minute weekly format demands a five-month commitment that may burden families facing transport or financial barriers. In addition, participation required active help-seeking, parental engagement, and regular attendance. As a result, families facing linguistic, socioeconomic, geographic, or psychosocial barriers may have been less likely to participate, potentially affecting the representativeness of the sample. To scale this model in resource-constrained settings, future research should explore lower-threshold adaptations, such as digital delivery, condensed formats, or task-sharing models with trained non-specialist community health workers. Finally, the modest and clinically heterogeneous sample limits generalizability, while also reflecting real-world public mental health settings.

## Conclusions

This pilot study offers preliminary support for the feasibility and clinical promise of a parent-only DBT skills group for families of high-risk adolescents. By strengthening parents’ coping, emotion regulation, validation, and crisis-response capacities, the intervention may reduce parental helplessness and support improvements in family functioning and adolescent clinical severity. However, less stable changes in family power struggles and emotion regulation difficulties suggest that broader interaction patterns may require continued reinforcement. In an overburdened public health system, this low-intensity group model may offer a scalable pathway for supporting adolescents through targeted work with their parents.

## Data Availability

The raw data supporting the conclusions of this article will be made available by the authors, without undue reservation.
