# Supplementary material for: Feasibility of a parent-only DBT skills group in public mental health services: a pilot implementation study
Source: Front Psychiatry. 2026 Aug 13;17:1888780. doi: 10.3389/fpsyt.2026.1888780 (PMC13518587; doi:10.3389/fpsyt.2026.1888780)
Supplement: Supplementary file 1 [file Table1.docx]

# **Supplementary Table 1.** DBT Skills Group for Parents: Session Plan

| Weeks | Topic | Content |
| --- | --- | --- |
| 1–2 | Orientation to Treatment | • What is DBT?  • Emotional regulation disorder overview  • Core DBT skills  • Bio-social model  • Core assumptions |
| 3–6 | Mindfulness | • Taking control  • Importance of mindfulness  • Three states of mind  • “What” skills  • “How” skills |
| 7–11 | Dialectics and Validation | • What is dialectics?  • Dialectics in practice  • Cognitive distortions  • What is validation?  • Self-validation  • Validating others |
| 12–15 | Emotional Regulation | • Functions of emotions  • Emotional development model  • ABC-PLEASE  • Fact-checking and opposite action |
| 16–17 | Distress Tolerance | • Purpose of tolerating painful emotions  • Distress tolerance skills  • Self-soothing with senses  • Improving the moment  • Pros and cons  • TIPP skills  • Distress-tolerance toolbox  • Radical acceptance |
| 18–19 | Interpersonal Effectiveness | • Clarifying goals and priorities  • GIVE  • DEAR MAN  • FAST |
| 20 | Final Meeting | • Summary and feedback |
